# Long-term incidence of first hospital-recorded atrial arrhythmias and all-cause mortality after percutaneous patent foramen ovale closure: a single-centre cohort linked to Czech nationwide administrative health data

**DOI:** 10.3389/fcvm.2026.1896548

**Published:** 2026-08-25

**Authors:** Martina Podolec, Anna Řeháková, Jiří Jarkovský, Aneta Dvořáková, Petr Volf, Petr Neužil, Martin Mates

**Affiliations:** 1 Department of Cardiology, 1st Medical Faculty of Charles University and Na Homolce Hospital, Motol and Homolka University Hospital, Homolka Campus, Prague, Czechia; 2 Institute of Health Information and Statistics of the Czech Republic, Prague, Czechia; 3 Institute of Biostatistics and Analyses, Masaryk University, Brno, Czechia; 4 Department of Internal Medicine, 2nd Medical Faculty of Charles University, Prague, Czechia

**Keywords:** administrative health data, atrial arrhythmia, atrial fibrillation, catheter ablation, patent foramen ovale, percutaneous closure, relative survival, stroke prevention

## Abstract

**Background:**

Percutaneous patent foramen ovale (PFO) closure is an established strategy for secondary prevention of embolic stroke of undetermined source (ESUS), but long-term arrhythmic and survival outcomes outside randomised trial populations remain incompletely characterised. Using nationwide Czech administrative health registries, we evaluated hospital-recorded atrial arrhythmias (AA), catheter ablation, antithrombotic therapy and mortality over 15 years.

**Methods:**

Consecutive patients undergoing percutaneous PFO closure at a single Czech tertiary referral centre between 1 January 2010 and 31 December 2024 were linked to nationwide Czech hospitalisation, pharmacy-dispensing and mortality databases. Time-to-event analyses used Kaplan–Meier and Fine–Gray competing-risks estimators; relative survival was estimated using the Pohar–Perme method against age-, sex- and calendar year-matched Czech population life tables.

**Results:**

A total of 715 procedures were performed in 707 patients [median age 49.4 years (IQR 41.7–59.0); 48.0% male]. Over a maximum follow-up of 14.9 years, 13 patients had a first clinically recognised, hospital-recorded AA (10-year cumulative incidence 2.9%, 95% CI 1.2%–4.6%). Five of the 13 events (38.5%) occurred within the first year, whereas 6 (46.2%) occurred beyond three years; affected patients were predominantly male with a high baseline cardiovascular risk burden. Post-procedural antithrombotic therapy was predominantly antiplatelet-based. The 10-year cumulative incidence of catheter ablation was 2.0% (95% CI 0.6%–3.3%). Thirty-four patients died (10-year all-cause mortality 7.5%, 95% CI 4.8–10.3%), mostly from neoplastic (35.3%) or cardiovascular (32.4%) causes. Relative survival remained close to unity and at no time point fell significantly below it (10-year 1.023, 95% CI 0.982–1.065), indicating no observed excess mortality relative to population life-table expectations.

**Conclusions:**

Clinically recognised, hospital-recorded AA events after PFO closure were uncommon. Because only 13 events were observed, their timing and association with cardiovascular risk factors are descriptive and hypothesis-generating, and the estimate does not capture total subclinical arrhythmic burden. No observed excess mortality was identified relative to population life-table expectations. These findings support prospective evaluation of risk-stratified rhythm surveillance.

## 1 Introduction

Patent foramen ovale (PFO) persists into adulthood in approximately one quarter of the general population and has been implicated in embolic stroke of undetermined source (ESUS), particularly in patients younger than 60 years (1, 2). Following publication of the pivotal randomised trials RESPECT, CLOSE and GORE-REDUCE, together with subsequent European and North American guideline updates, percutaneous device closure has become an established strategy for secondary stroke prevention in carefully selected patients (3–7).

Although PFO closure reduces the risk of recurrent ischaemic stroke, the procedure is associated with recognised complications. Early post-procedural atrial fibrillation (AF), atrial flutter (AFL) and atrial tachycardia (AT)—collectively referred to here as atrial arrhythmias (AA)—are now well described. Reported incidences in randomised trials range from approximately 4% to 6% within 45 days, whereas prolonged-monitoring studies in selected populations have identified substantially higher rates (8–14). Whether this early arrhythmic signal represents a transient peri-procedural phenomenon or reflects a more durable arrhythmogenic atrial substrate has remained debated for more than a decade (15–18).

Long-term evidence derives from both randomised trials and large administrative-data cohorts. Extended follow-up from CLOSE, RESPECT and GORE-REDUCE suggests that the absolute long-term risk of AA attributable to device closure remains modest, although interpretation is limited by small event numbers (3–5, 19, 20). Large registry-based studies—including nationwide Danish and Ontario population linkages—have extended follow-up to more than a decade and generally reported cumulative AF incidences approaching background population rates (21–23). Nevertheless, substantial heterogeneity persists across studies in design, surveillance intensity and patient selection, and contemporary Central European data combining detailed single-centre procedural information with complete nationwide registry follow-up remain limited.

Whether percutaneous PFO closure is associated with excess long-term mortality also remains uncertain. Many published cohorts report observed survival without benchmarking against expected population mortality, limiting interpretation. Relative survival analysis, in which observed survival is compared with matched population life tables, offers a methodologically rigorous approach to this question (24) and has not, to our knowledge, previously been applied to a national PFO closure cohort.

We therefore undertook a retrospective single-centre cohort study of patients undergoing percutaneous PFO closure between 2010 and 2024 at a high-volume Czech tertiary referral centre, with deterministic linkage through the national personal identifier to nationwide hospitalisation, pharmacy-dispensing and mortality registries. Our objectives were three-fold: (i) to quantify the long-term incidence of hospital-recorded AA events and subsequent catheter ablation for AA; (ii) to describe the demographic and comorbidity profile of patients developing AA together with patterns of dispensed antithrombotic therapy; and (iii) to compare observed all-cause and cause-specific mortality with that expected in the matched Czech general population using the Pohar–Perme relative survival estimator.

## 2 Materials and methods

### 2.1 Study design and setting

This was a single-centre retrospective analysis of a prospectively maintained interventional registry, supplemented by deterministic linkage to nationwide Czech administrative health databases. All percutaneous PFO closures performed at the Cardiology Department of Motol and Homolka University Hospital, Homolka Campus, Prague, Czech Republic, between 1 January 2010 and 31 December 2024 were eligible. The Homolka Campus of Motol and Homolka University Hospital is one of the highest-volume structural interventional centres in the Czech Republic and serves as a tertiary referral site for cryptogenic stroke and ESUS.

### 2.2 Data sources

Three data sources were used.

First, the prospectively maintained hospital interventional registry provided procedural information (procedure date and in-hospital adverse events), together with demographic and baseline clinical characteristics. Device-level variables were not included in the dataset transferred for linkage and analysis.

Second, the National Registry of Reimbursed Health Services (Národní registr hrazených zdravotních služeb, NRHZS), administered by the Institute of Health Information and Statistics of the Czech Republic (Ústav zdravotnických informací a statistiky ČR, ÚZIS ČR), provided hospital admission records, hospital-based outpatient specialist encounters, procedure codes from the Czech National List of Healthcare Services, ICD-10 diagnoses and dispensed-medication records mapped to the Anatomical Therapeutic Chemical (ATC) classification.

Third, the Czech Statistical Office database of deceased persons (Český statistický úřad, ČSÚ) provided the date and primary cause of death classified according to ICD-10 chapters. Linkage across these data sources was performed deterministically using the Czech national personal identifier (rodné číslo).

### 2.3 Study population

Consecutive adult patients (aged ≥18 years) who underwent successful percutaneous PFO closure during the study period were eligible. Absence of previously documented AF, AFL or AT was a prerequisite for the closure indication: patients with a known history of AA were not referred for closure, and all patients underwent pre-closure rhythm assessment—including at least 24-hour Holter electrocardiographic monitoring (with prolonged ambulatory monitoring increasingly employed in more recent years)—required to be free of AA; implantable cardiac monitors were not routinely used before closure. No patient was excluded from the cohort on administrative grounds; absence of documented AA was a clinical prerequisite for the closure indication, although a single short-duration Holter cannot fully exclude subclinical paroxysmal arrhythmia. Indications for closure followed institutional protocols aligned with contemporaneous European Society of Cardiology (ESC) recommendations and the European position paper on PFO management (6). The predominant indication was cryptogenic stroke or transient ischaemic attack (TIA) consistent with paradoxical embolism in patients with a clinically and anatomically appropriate PFO. The indication was neurology-led: closure was undertaken on the recommendation of the treating neurologist following the cryptogenic stroke/TIA workup, which commonly included transcranial Doppler and transoesophageal echocardiographic assessment. No prespecified minimum microbubble count was applied as an independent mandatory criterion for closure; the decision was based on the overall neurological assessment, demonstration of a clinically relevant right-to-left shunt and anatomical feasibility. Specific echocardiographic high-risk septal features (for example a large shunt or atrial septal aneurysm) were not applied as mandatory criteria for closure at our centre, and no strict upper age limit was applied; in selected older patients, including those older than 65 years, closure was performed when recommended by the neurologist and considered anatomically feasible, reflecting a multidisciplinary, individualised approach. Exact patient-level counts according to indication subtype were not available in the dataset transferred for linkage and analysis. Repeat procedures in the same patient, for example for residual right-to-left shunt, were counted as separate procedures but contributed only once to patient-level analyses.

### 2.4 Procedural technique

Procedures were performed under fluoroscopic and transoesophageal or intracardiac echocardiographic guidance in the cardiac catheterisation laboratory using femoral venous access. Devices implanted during the study period included the Amplatzer PFO Occluder and Amplatzer Talisman (Abbott), the Cardia Ultrasept (Cardia Inc.), the Occlutech Figulla Flex (Occlutech) and the LifeTech CeraFlex (LifeTech). Device selection was guided by individual septal anatomy. In contemporary practice, the Cardia Ultrasept, Figulla Flex and CeraFlex devices are used predominantly, whereas Amplatzer devices are used less frequently.

Peri-procedural anticoagulation and antibiotic prophylaxis followed institutional protocols and contemporaneous best practice. The institutional post-closure antithrombotic protocol comprised dual antiplatelet therapy (DAPT) for six months. During the earlier part of the study period, antiplatelet therapy was intended to be discontinued thereafter; in practice, single antiplatelet therapy (SAPT; acetylsalicylic acid or clopidogrel) was commonly continued at the recommendation of the referring neurologist. Following publication of the pivotal randomised trials and the 2019 European position paper (6), the institutional protocol was formalised as DAPT for six months followed by SAPT—preferably acetylsalicylic acid—for at least five years, unless a specific indication for oral anticoagulation or clopidogrel was present. The subsequent European Stroke Organisation guideline (25) separately advises against long-term anticoagulation after PFO-associated stroke unless another indication for anticoagulation is present. Long-term antiplatelet persistence was not assessed in this study.

### 2.5 Outcomes and definitions

The principal outcome was the first hospital-recorded AA after closure, defined as the first inpatient admission, day-case attendance or hospital-based outpatient specialist encounter at which AF, AFL or AT/supraventricular tachycardia was recorded as a primary or secondary diagnosis after the index PFO closure. A qualifying encounter therefore represents a clinically recognised arrhythmic episode prompting hospital-based evaluation and/or treatment. This includes inpatient events and hospital-based outpatient events, such as acute ambulatory electrical cardioversion or presentation to an emergency department or acute cardiology clinic. Electrical cardioversion does not have a discrete procedure code in the Czech National List of Healthcare Services and, unlike catheter ablation, could not be identified separately. The endpoint was operationally defined as the first hospital-recorded AA after closure in a patient without clinically documented AA before closure (see Materials and methods, section 2.3). Because a single short-duration Holter cannot exclude subclinical paroxysmal AA, the endpoint is interpreted as the first hospital-recorded AA after closure rather than as strictly incident disease. Time to event was calculated from the date of PFO closure to the date of the first qualifying encounter. The ICD-10 codes used to define AA are listed in Table 1.

**Table 1:** Annual distribution of first hospital-recorded atrial arrhythmias by ICD-10 code in patients undergoing PFO closure, 2010–2024.

Year of PFO	n	I48.0	I48.1	I48.3	I47.1	≥1 code
2010	17	–	–	–	–	0
2011	26	–	–	–	–	0
2012	15	–	–	–	–	0
2013	35	–	–	–	–	0
2014	48	–	–	1	–	1
2015	49	1	1	–	–	2
2016	46	1	1	–	–	2
2017	49	–	1	–	1	2
2018	59	–	–	–	1	1
2019	73	1	–	–	–	1
2020	62	1	–	–	–	1
2021	62	1	1	–	–	2
2022	55	1	–	–	–	1
2023	63	–	–	–	–	0
2024	48	–	–	–	–	0
**Total**	**707**	**6**	**4**	**1**	**2**	**13**

Counts indicate the number of unique patients who had a first hospital-recorded AA with each ICD-10 code after PFO closure. No qualifying events were recorded for codes I48.2, I48.4, I48.9 or I47.9 during the study period.

ICD-10 codes: I47.1, supraventricular tachycardia; I47.9, paroxysmal tachycardia, unspecified; I48.0, paroxysmal atrial fibrillation; I48.1, persistent atrial fibrillation; I48.2, chronic atrial fibrillation; I48.3, typical atrial flutter; I48.4, atypical atrial flutter; I48.9, atrial fibrillation and flutter, unspecified.

Bold values indicate totals.

Catheter ablation was identified using procedure codes from the Czech National List of Healthcare Services corresponding to electrophysiological ablation of AA: 17312 (catheter ablation for atrial fibrillation), 17308 (catheter ablation for supraventricular tachycardia or atrial flutter) and 17610 (electrophysiological catheter ablation of cardiac arrhythmia). The procedure codes used to identify catheter ablation are also listed in Table 2.

**Table 2:** Kaplan–Meier cumulative incidence of catheter ablation after PFO closure (*n* = 707).

Follow-up	Ablation, % (95% CI)	Patients at risk
Baseline	0.0% (0.0%–0.0%)	707
30 days	0.0% (0.0%–0.0%)	—
1 year	0.3% (0.0%–0.7%)	—
2 years	0.3% (0.0%–0.7%)	588
3 years	0.6% (0.0%–1.3%)	—
4 years	1.0% (0.2%–1.9%)	462
5 years	1.3% (0.3%–2.2%)	—
6 years	1.5% (0.5%–2.6%)	324
7 years	1.5% (0.5%–2.6%)	—
8 years	2.0% (0.6%–3.3%)	217
9 years	2.0% (0.6%–3.3%)	—
10 years	2.0% (0.6%–3.3%)	128

CI, confidence interval. Cumulative incidence estimated from Kaplan–Meier with Greenwood-derived 95% CIs.

Numbers at risk are reported at baseline and at two-year intervals. Em dashes indicate time points for which numbers at risk were not separately reported.

Procedure codes: 17312, catheter ablation for atrial fibrillation; 17308, catheter ablation for supraventricular tachycardia/atrial flutter; 17610, electrophysiological catheter ablation of cardiac arrhythmia.

All-cause mortality was defined as death from any cause, ascertained from the ČSÚ database. Cause-specific mortality was classified according to the primary cause of death by ICD-10 chapter. Outcomes were ascertained through 31 December 2024.

### 2.6 Baseline comorbidities and concomitant pharmacotherapy

Baseline comorbidities were ascertained from administrative records covering the calendar year of PFO closure and all preceding years available in NRHZS. Diabetes mellitus (DM) was defined as any record of ICD-10 codes E10–E14 and/or dispensing of an antidiabetic agent. Arterial hypertension (HTN) was defined as ICD-10 code I10 and/or dispensing of an antihypertensive agent. Hyperlipidaemia (HLP) was defined operationally as documented dispensing of a lipid-modifying agent in the year of closure or earlier; this pragmatic definition was preferred over diagnosis coding because lipid disorders are systematically under-coded in inpatient discharge records.

Antithrombotic therapy was characterised through pharmacy-dispensing records within 90 days after the index procedure. The agents considered comprised antiplatelet drugs (clopidogrel, acetylsalicylic acid, prasugrel and ticagrelor), vitamin K antagonists (warfarin), heparins, fondaparinux and direct oral anticoagulants (dabigatran, rivaroxaban, apixaban and edoxaban, DOACs). Statins and combination lipid-modifying preparations were identified within the corresponding ATC subgroups.

### 2.7 Statistical analysis

Continuous variables were summarised as mean ± standard deviation or as median with interquartile range, depending on distributional properties. Categorical variables were expressed as counts and percentages. The cumulative incidences of AA, catheter ablation and all-cause mortality were estimated by the Kaplan–Meier method, with 95% confidence intervals (CIs) derived using the Greenwood formula and the complementary log-log transformation. Patients without an event were censored at the date of death (where applicable, for non-mortality endpoints) or at 31 December 2024. Because death during follow-up acts as a competing event for AA, the cumulative incidence of AA was additionally estimated using a Fine–Gray competing-risks model as a sensitivity analysis. Numbers at risk are reported at baseline and at two-year intervals for the Kaplan–Meier analyses.

Relative survival was estimated using the Pohar–Perme non-parametric estimator (24), which provides a consistent estimator of net survival in the absence of cause-of-death information. Expected survival was computed from age-, sex- and calendar-year-stratified life tables for the Czech Republic published by ČSÚ. A relative survival ratio of 1.0 indicates that observed survival is equal to the matched-population expectation; values consistently below 1.0 imply excess mortality. Confidence intervals were obtained using the log-transformation method.

Given the modest number of arrhythmic events (*n* = 13), multivariable modelling was not pursued; demographic and comorbidity profiles of patients developing arrhythmia are presented descriptively. Accordingly, all comparisons between patients who did and did not develop AA, and all statements concerning the temporal distribution of events, are descriptive and hypothesis-generating only and should not be interpreted as evidence of independent association or causation. No imputation of missing data was performed. All analyses were performed using standard statistical software at ÚZIS ČR. Data extraction, aggregation, and calculation of descriptive statistics were performed using SQL queries. Datasets for survival analyses were prepared in IBM SPSS Statistics, version 29.0.1.1. Statistical analyses and data visualisation were conducted in R statistical software (R Foundation for Statistical Computing, Vienna, Austria), version 3.6.3 (2020-02-29). The following R packages were used: ranger, dplyr, ggfortify, coin, survival, relsurv, readxl, survminer, lubridate, ggplot2, and readr.

### 2.8 Ethics and data governance

The study was conducted in accordance with the Declaration of Helsinki and Czech Act No. 372/2011 Coll. on Health Services. Institutional procedural data were transferred to ÚZIS ČR for deterministic linkage with national administrative databases and statistical analysis and were pseudonymised prior to analysis. The use of pseudonymised national administrative data for analytical purposes is permitted under Czech legislation and GDPR Article 9(2)(j). Given the retrospective nature of the study and the exclusive use of pseudonymised registry data, formal ethics committee approval and individual informed consent were not required under applicable Czech legislation.

## 3 Results

### 3.1 Cohort and procedural characteristics

**Figure 1:**
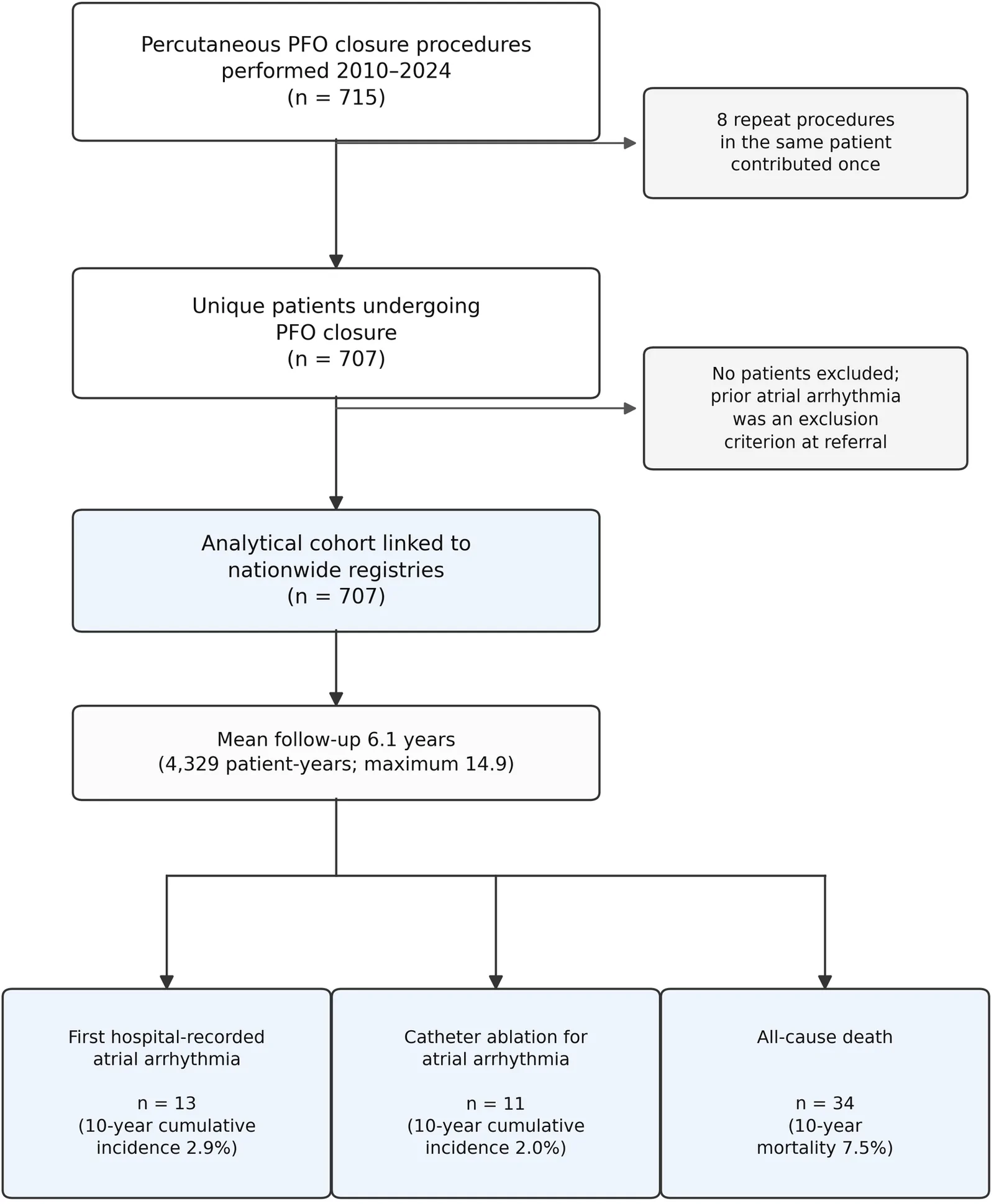
Derivation of the study cohort and outcomes. Flow of participants from all percutaneous PFO closure procedures performed between 2010 and 2024 to the analytical cohort and the three study outcomes. Repeat procedures in the same patient contributed once to patient-level analyses. Patients with previously documented atrial arrhythmia were not referred for closure and were therefore excluded at the referral stage rather than from the linked dataset.

**Figure 2:**
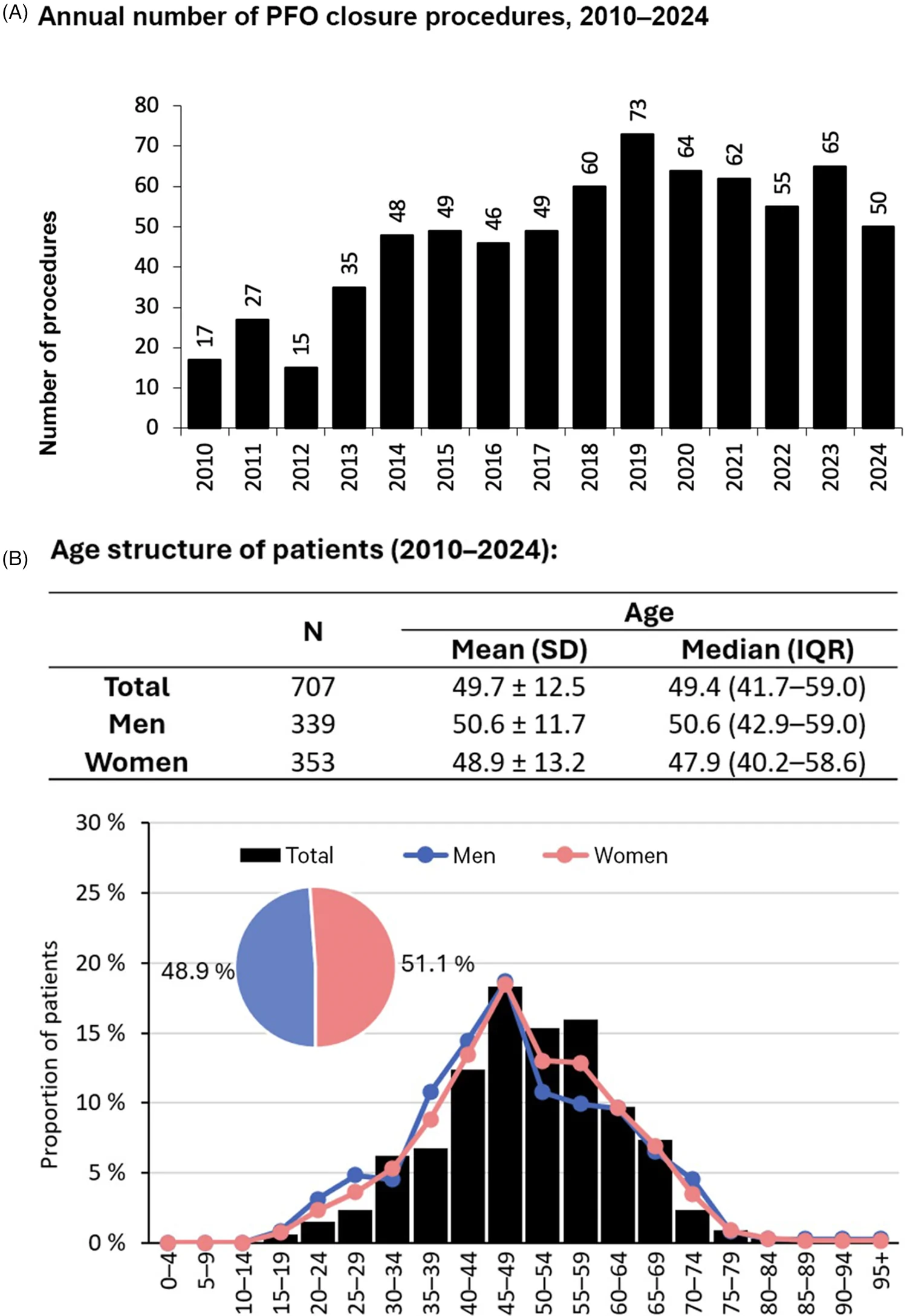
Cohort characteristics and procedural volume. **(A)** Number of procedures: annual number of percutaneous PFO closures between 2010 and 2024. **(B)** Age and sex distribution of the study cohort. Sex percentages are calculated among patients with documented sex (*n* = 692); sex was undocumented in 15 patients.

Between 1 January 2010 and 31 December 2024, 715 percutaneous PFO closure procedures were performed in 707 unique patients (Figure 1). Annual procedural volume rose from 17 cases in 2010 to a sustained level of 48–73 cases per year from 2017 onwards, reflecting growing referral following publication of the 2017 randomised trials. Median age was 49.4 years (IQR 41.7–59.0), and mean age was 49.7 ± 12.5 years. Men accounted for 48.0% of patients (*n* = 339, mean age 50.6 ± 11.7 years) and women for 49.9% (*n* = 353, mean age 48.9 ± 13.2 years). Sex was not documented in 15 records (2.1%). Baseline characteristics are summarised in Table 3. Annual procedural volume and the age distribution are illustrated in Figure 2.

**Table 3:** Baseline demographic and clinical characteristics of the study cohort.

Characteristic	Value
Procedures, n	715
Patients, n	707
Age at procedure, years—mean ± SD	49.7 ± 12.5
Age at procedure, years—median (IQR)	49.4 (41.7–59.0)
Male sex, *n* (%)	339 (48.0%)
Female sex, *n* (%)	353 (49.9%)
Sex not documented, *n* (%)	15 (2.1%)
Arterial hypertension, *n* (%)	440 (62.2%)
Hyperlipidaemia, *n* (%)	493 (69.7%)
Diabetes mellitus, *n* (%)	106 (15.0%)

IQR, interquartile range; SD, standard deviation. Comorbidities ascertained from NRHZS in the year of PFO closure and earlier (see Materials and methods, section 2.6).

### 3.2 Comorbidity prevalence across the study period

**Figure 3:**
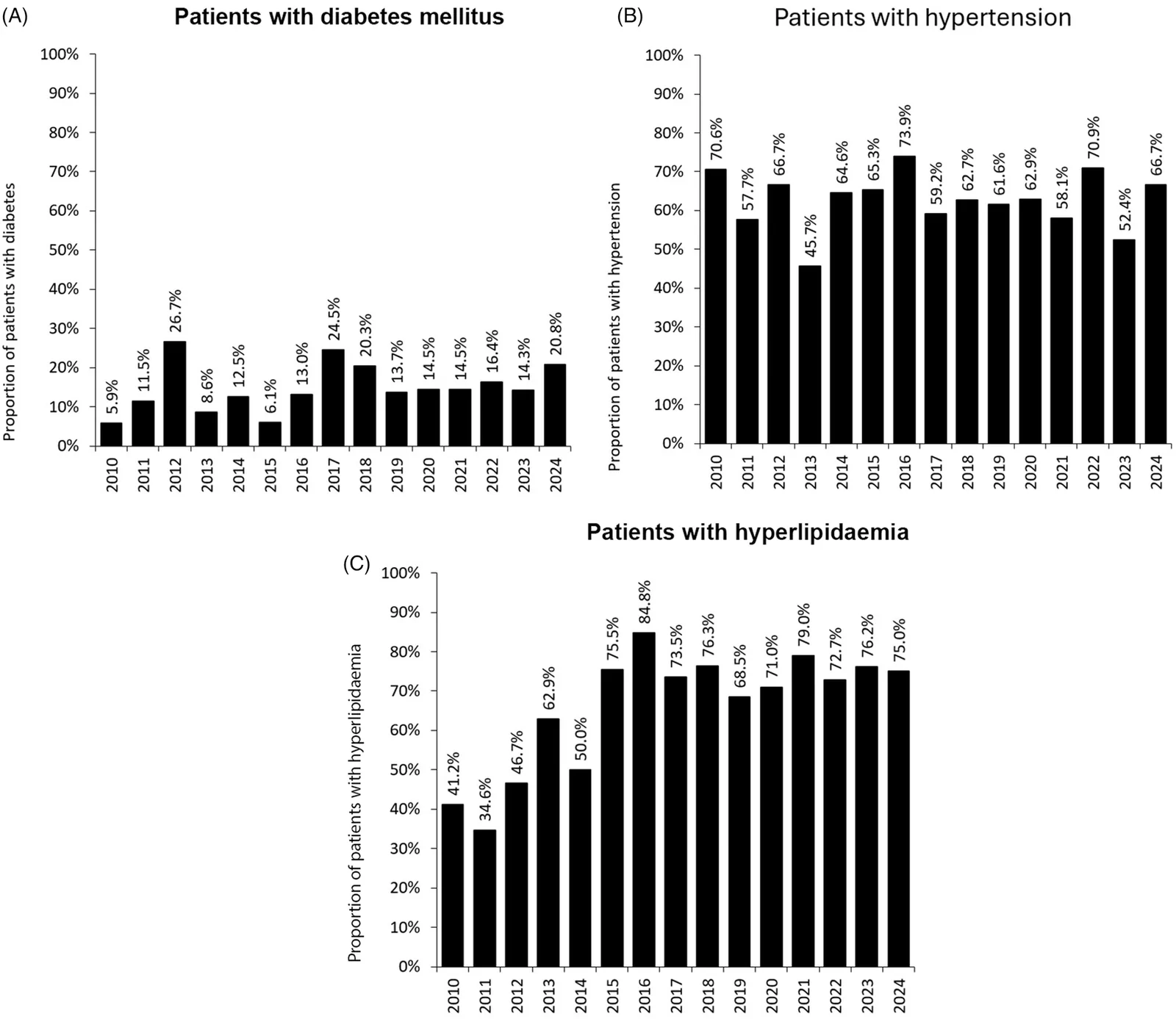
Temporal trends in cardiovascular comorbidity prevalence among patients undergoing percutaneous PFO closure between 2010 and 2024. **(A)** Prevalence of diabetes mellitus, defined by ICD-10 codes E10–E14 and/or dispensing of antidiabetic medication (ATC group A10). **(B)** Prevalence of arterial hypertension, defined by ICD-10 code I10 and/or dispensing of antihypertensive medication (ATC groups C02, C03, C07–C09). **(C)** Prevalence of hyperlipidaemia, operationally defined by dispensing of lipid-modifying therapy (selected ATC C10 codes) in the year of PFO closure or earlier. Three-panel figure demonstrating annual prevalence trends of diabetes mellitus, arterial hypertension and hyperlipidaemia among patients undergoing percutaneous patent foramen ovale closure between 2010 and 2024.

Cardiovascular comorbidities were common among patients referred for PFO closure. The annual prevalence of DM, HTN and HLP varied across the study period and is summarised in Supplementary Table S1 and depicted in Figure 3.

### 3.3 Antithrombotic pharmacotherapy

Antithrombotic therapy within 90 days after percutaneous PFO closure was predominantly antiplatelet-based (Supplementary Table S2). Acetylsalicylic acid and clopidogrel were the most frequently dispensed agents throughout the study period, consistent with the expected use of dual antiplatelet therapy after PFO closure. Oral anticoagulation was used less frequently and mainly in selected patients. Over time, anticoagulant prescribing shifted from warfarin towards DOACs. Detailed annual dispensing patterns for acetylsalicylic acid, clopidogrel, warfarin and DOACs are shown in Supplementary Table S2.

### 3.4 Incidence and clinical spectrum of atrial arrhythmias

**Figure 4:**
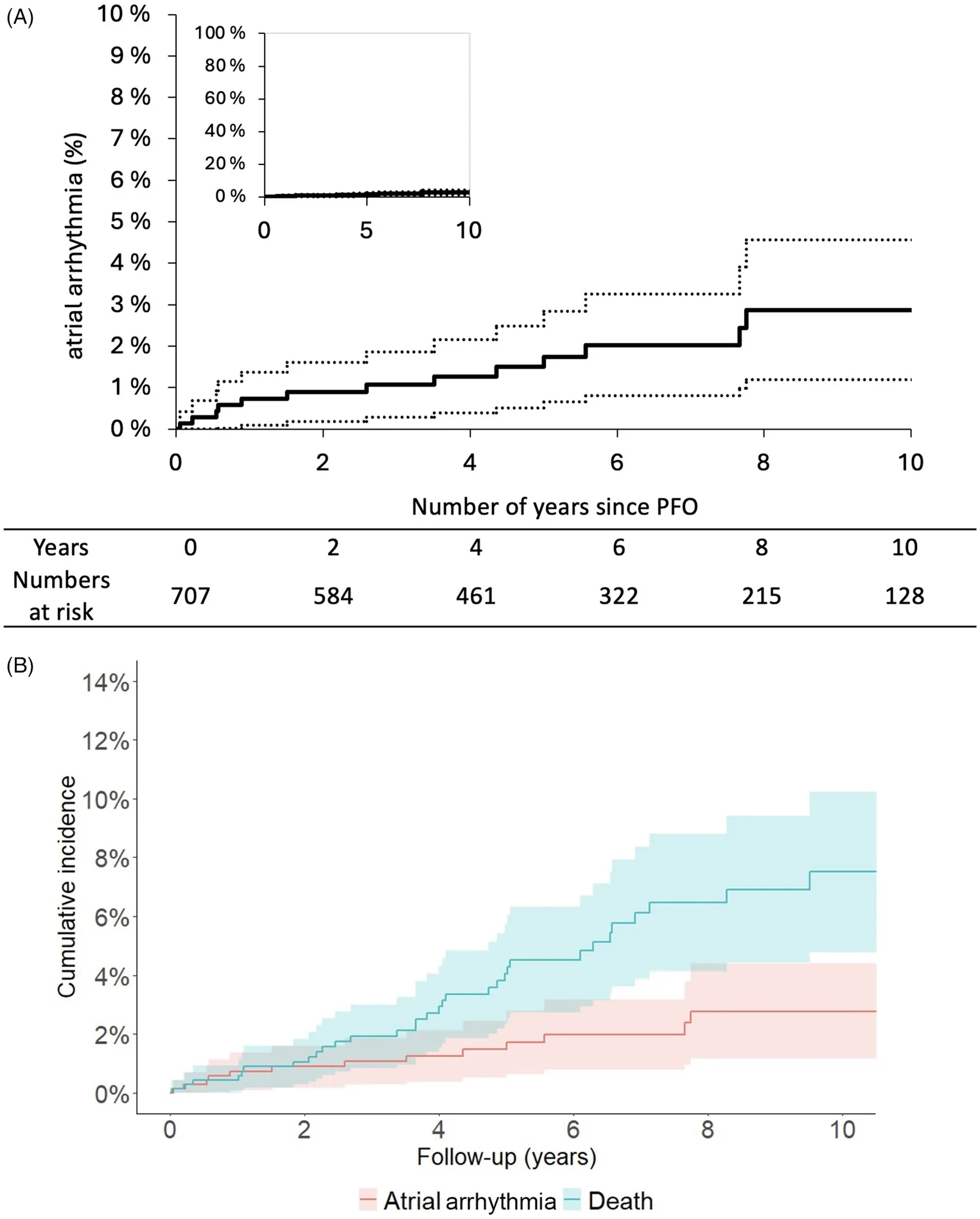
Cumulative incidence of first hospital-recorded atrial arrhythmia after percutaneous PFO closure. **(A)** Kaplan–Meier cumulative incidence, with numbers at risk shown beneath the *x*-axis. **(B)** Fine–Gray cumulative incidence function accounting for death as a competing event. Cumulative incidence functions estimated using the Fine–Gray competing-risks model in the full study cohort (*n* = 707), showing atrial arrhythmia (event of interest) and death (competing event); shaded areas represent 95% confidence intervals. Patients were followed from the date of percutaneous PFO closure until the occurrence of atrial arrhythmia, death, or the end of follow-up on 31 December 2024.

Across a total observation time of 4,329 patient-years (mean follow-up 6.1 years per patient; range 0–14.9 years), 13 of 707 patients (1.84%) had a first hospital-recorded AA after closure. Follow-up duration varied markedly by procedure year: patients treated in the earliest years contributed up to 14.9 years, whereas those treated in 2023–2024 contributed only months. Long-term incidence estimates are therefore informed predominantly by the earlier procedural cohorts. The annual distribution of cases, stratified by ICD-10 code, is given in Table 1. The Kaplan–Meier cumulative incidence of first hospital-recorded AA was 0.7% at 1 year (95% CI 0.1%–1.4%), 1.5% at 5 years (95% CI 0.5%–2.5%) and 2.9% at 10 years (95% CI 1.2%–4.6%); 707 patients were at risk at baseline and 128 at 10 years (Table 4, Figure 4A). The cumulative incidence estimate exceeds the crude proportion (1.84%) because it accounts for censoring and variable follow-up duration. In the Fine–Gray competing-risks sensitivity analysis, with death treated as a competing event, the corresponding 10-year cumulative incidence was 2.8% (95% CI 1.2%–4.4%), essentially unchanged (Table 4, Figure 4B). Of the 13 events, 11 were atrial fibrillation or atrial flutter (paroxysmal atrial fibrillation [I48.0], *n* = 6; persistent atrial fibrillation [I48.1], *n* = 4; typical atrial flutter [I48.3], *n* = 1) and 2 were supraventricular tachycardia (I47.1); no less-specific codes contributed events. The supraventricular tachycardia code (I47.1) was retained because administrative coding of paroxysmal atrial tachyarrhythmias is physician-dependent and may be applied non-specifically; its inclusion avoids missing miscoded atrial fibrillation or flutter, at the cost of a possible marginal overestimate of these entities.

**Table 4:** Kaplan–Meier cumulative incidence of the first hospital-recorded atrial arrhythmia after PFO closure (*n* = 707).

Follow-up	Cumulative incidence of AA, % (95% CI)	Patients at risk
Baseline	0.0% (0.0%–0.0%)	707
30 days	0.1% (0.0%–0.4%)	—
1 year	0.7% (0.1%–1.4%)	—
2 years	0.9% (0.2%–1.6%)	584
3 years	1.1% (0.3%–1.9%)	—
4 years	1.3% (0.4%–2.2%)	461
5 years	1.5% (0.5%–2.5%)	—
6 years	2.0% (0.8%–3.3%)	322
7 years	2.0% (0.8%–3.3%)	—
8 years	2.9% (1.2%–4.6%)	215
9 years	2.9% (1.2%–4.6%)	—
10 years	2.9% (1.2%–4.6%)	128

AA, atrial arrhythmia; CI, confidence interval. Cumulative incidence of the first hospital-recorded atrial arrhythmia after PFO closure (ICD-10 codes I47.1, I47.9 and I48.0–I48.9; only I47.1, I48.0, I48.1 and I48.3 contributed events), estimated by the Kaplan–Meier method with Greenwood-derived 95% CIs.

Numbers at risk are reported at baseline and at two-year intervals. Em dashes indicate time points for which numbers at risk were not separately reported.

In a Fine–Gray competing-risks analysis accounting for death as a competing event, the corresponding cumulative incidence was 0.7% (95% CI 0.1%–1.4%) at 1 year and 2.8% (95% CI 1.2%–4.4%) at 10 years.

Qualifying events were distributed across procedure cohorts from 2014 to 2022. No events were recorded among patients treated in 2010–2013. The reason cannot be determined from the available data; smaller cohort size, chance and differences in recorded baseline characteristics may have contributed. No events were recorded among patients treated in 2023–2024, consistent with their limited follow-up.

### 3.5 Demographic and comorbidity profile of patients developing atrial arrhythmia

Patients with a first hospital-recorded AA differed descriptively from the parent cohort in sex distribution and baseline cardiovascular risk profile (Table 5). Of 13 affected patients, 9 were men (69.2%) and 4 were women (30.8%), in contrast with the 48.0%/49.9% sex distribution of the parent cohort. Higher observed proportions of cardiovascular comorbidities were recorded among patients who developed AA: HTN was present in 11 of 13 (84.6%) compared with 62.2% in the parent cohort, HLP in 12 of 13 (92.3%) compared with 69.7%, and DM in 3 of 13 (23.1%) compared with 15.0%. This pattern of male predominance, HTN and HLP mirrors the established cardiovascular risk-factor profile associated with AA in the general population and is examined further in section 4.4.

**Table 5:** Sex distribution and cardiovascular comorbidities of patients who developed a first hospital-recorded atrial arrhythmia after PFO closure (*n* = 13).

Year of PFO	AA cases	Men	Women	DM	HTN	HLP
2014	1	0	1	0	1	1
2015	2	2	0	0	2	2
2016	2	0	2	1	2	2
2017	2	1	1	1	1	2
2018	1	1	0	1	1	1
2019	1	1	0	0	1	0
2020	1	1	0	0	1	1
2021	2	2	0	0	1	2
2022	1	1	0	0	1	1
**Total**	**13**	**9 (69.2%)**	**4 (30.8%)**	**3 (23.1%)**	**11 (84.6%)**	**12 (92.3%)**

AA, atrial arrhythmia identified by ICD-10 codes I47.1, I47.9 and I48.0–I48.9; DM, diabetes mellitus; HLP, hyperlipidaemia; HTN, arterial hypertension. Comorbidities were ascertained in the year of PFO closure and earlier (see Materials and methods, section 2.6).

Bold values indicate totals.

### 3.6 Temporal pattern of atrial arrhythmias after closure

The temporal distribution of events is summarised in Table 6. Because the interval categories differ in length, the proportions are descriptive and not directly comparable. Two of 13 events (15.4%) occurred within the first 90 days—the conventional window for peri-procedural arrhythmia—and three further events (23.1%) between 90 days and 12 months. In contrast, eight of 13 events (61.5%) occurred more than one year after closure, including six events (46.2%) beyond three years. The longest observed interval was 9.7 years between closure and the first hospital-recorded AA episode. This pattern shows that some clinically recognised AA events requiring hospital-based evaluation were recorded several years after closure and were not confined to the immediate post-procedural period.

**Table 6:** Temporal distribution of the first hospital-recorded atrial arrhythmias after PFO closure (*n* = 13).

Interval from PFO closure to first arrhythmia	*n* (%)
≤ 90 days (peri-procedural window)	2 (15.4%)
> 90 days to 1 year	3 (23.1%)
> 1 year to 3 years	2 (15.4%)
> 3 years	6 (46.2%)
**Total**	**13** (**100%)**

Interval categories differ in width; counts are therefore descriptive and not directly comparable.

Bold values indicate totals.

### 3.7 Catheter ablation of atrial arrhythmias

**Figure 5:**
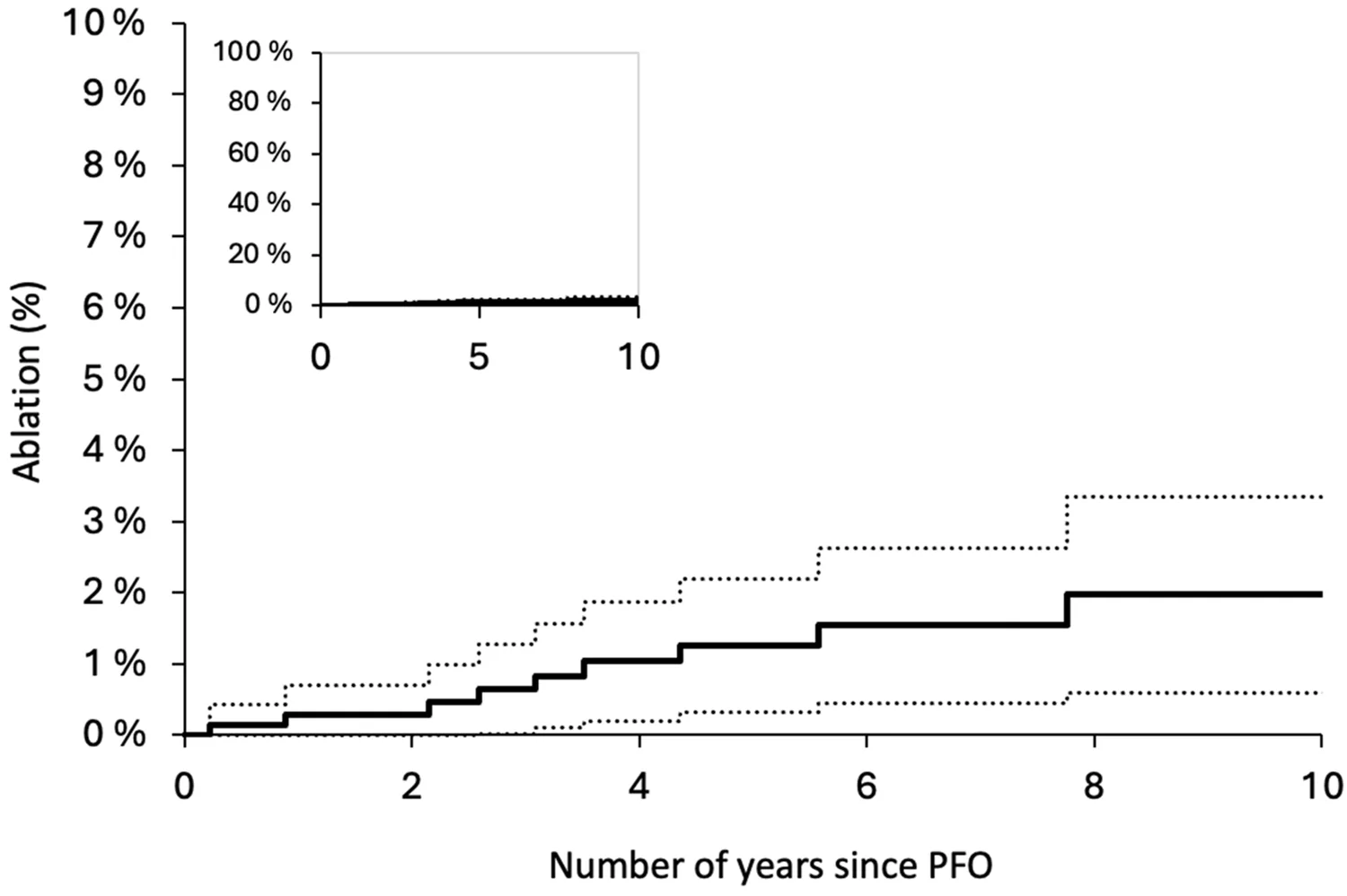
Kaplan–Meier cumulative incidence of catheter ablation following percutaneous PFO closure. Cumulative incidence of catheter ablation for atrial tachyarrhythmia after PFO closure in the study cohort (*n* = 707). Time-to-event analysis was performed using the Kaplan–Meier method. Follow-up was calculated from the date of percutaneous PFO closure to the date of first catheter ablation. Patients without a recorded ablation procedure were censored at the date of death or on 31 December 2024.

Catheter ablation for atrial tachyarrhythmia was performed in a small minority of patients during follow-up. Ablation was recorded in 11 of 707 patients (1.6%): code 17312 (ablation for atrial fibrillation) in 10 patients and code 17308 (ablation for supraventricular tachycardia or atrial flutter) in 2, with both codes recorded in one patient; code 17610 was not recorded. The Kaplan–Meier cumulative incidence of ablation increased from 0.3% at 1 year (95% CI 0.0%–0.7%) to 1.3% at 5 years (95% CI 0.3%–2.2%) and reached 2.0% at 10 years (95% CI 0.6%–3.3%); no further ablation procedures were observed beyond 8 years (Table 2, Figure 5). A total of 707 patients were at risk at baseline and 128 at 10 years. All catheter ablations were performed at our centre using transseptal access adjacent to, rather than directly through, the closure device. In no case was the implanted closure device documented to compromise transseptal puncture, consistent with feasibility data reported for both early-generation and contemporary devices (26, 27).

### 3.8 All-cause mortality and relative survival

**Figure 6:**
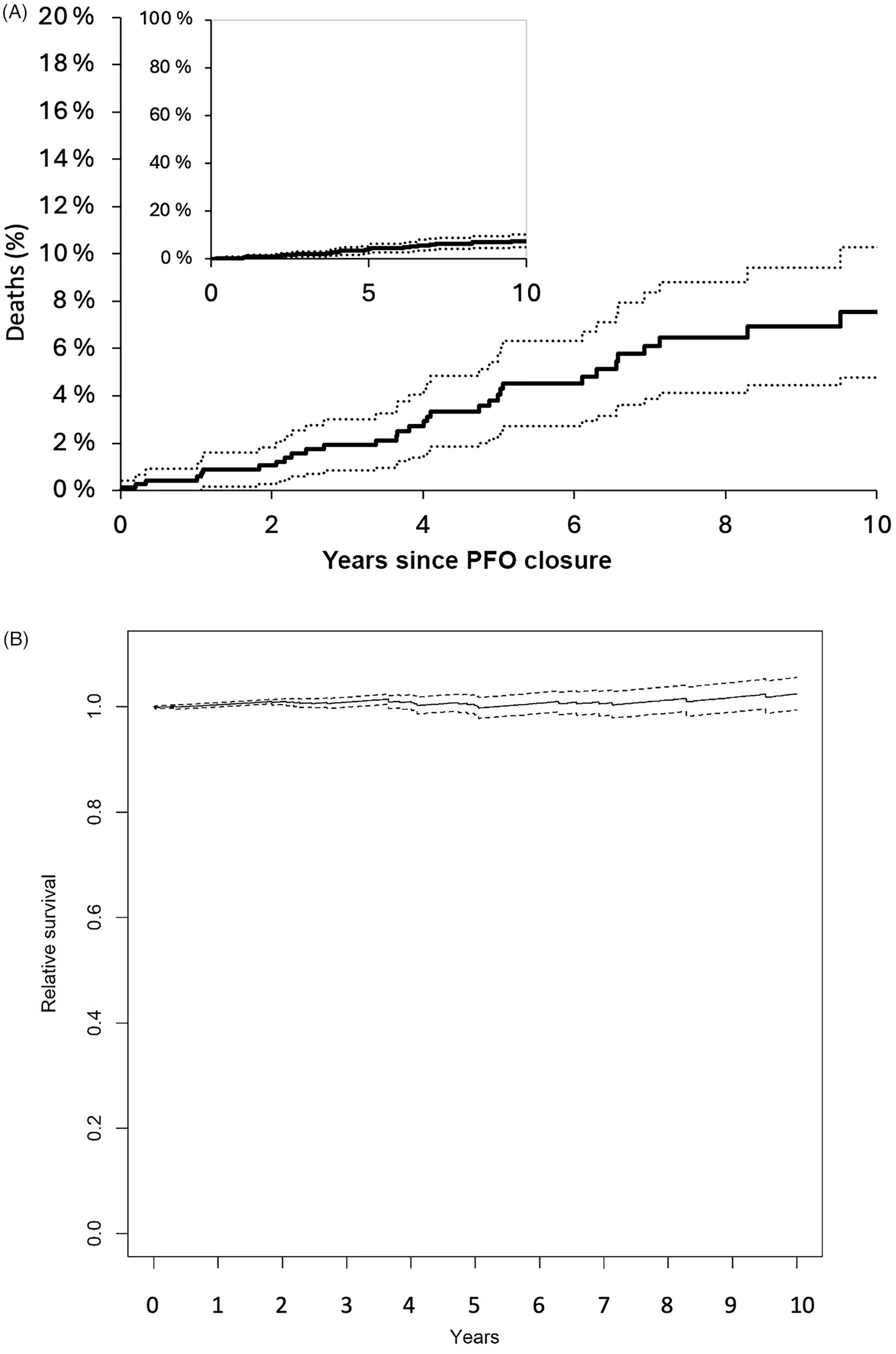
Long-term survival following percutaneous PFO closure. **(A)** Observed cumulative all-cause mortality estimated using the Kaplan–Meier method. **(B)** Net survival relative to the contemporaneous Czech general population estimated using the Pohar–Perme method. Survival analyses were performed in the full study cohort (*n* = 707). For Kaplan–Meier analysis, patients were followed from the date of percutaneous PFO closure until death or administrative censoring on 31 December 2024. Net survival was estimated using the Pohar–Perme method with age-, sex- and calendar year-matched Czech population life tables.

By 31 December 2024, 34 of 707 patients had died (4.8% crude mortality). The Kaplan–Meier cumulative incidence of all-cause death was 0.4% at 1 year (95% CI 0.0%–0.9%), 4.0% at 5 years (95% CI 2.4%–5.7%) and 7.5% at 10 years (95% CI 4.8–10.3%). Relative survival, estimated by the Pohar–Perme method against contemporaneous Czech population life tables, was close to unity throughout follow-up. The point estimate was marginally below unity only at 30 days (0.999) and at or above unity thereafter. The 95% CI lay entirely above unity at 1 and 2 years (1.008, 95% CI 1.003–1.013 and 1.008, 95% CI 1.002–1.014, respectively)—consistent with slightly better-than-expected survival in a population selected as fit for intervention—whereas at all other time points, including 10 years (1.023, 95% CI 0.982–1.065), the 95% CI included unity. At no time point was relative survival significantly below unity, indicating no observed excess mortality relative to the matched general population (Table 7, Figure 6).

**Table 7:** Observed all-cause mortality (Kaplan–Meier) and relative survival (Pohar–Perme) after PFO closure (*n* = 707).

Follow-up	Cumulative mortality, % (95% CI)	Relative survival (95% CI)	Patients at risk
Baseline	0.0% (0.0%–0.0%)	1.000 (1.000–1.000)	707
30 days	0.1% (0.0%–0.4%)	0.999 (0.996–1.001)	—
1 year	0.4% (0.0%–0.9%)	1.008 (1.003–1.013)	—
2 years	1.1% (0.3%–1.8%)	1.008 (1.002–1.014)	590
3 years	1.9% (0.8%–3.0%)	1.006 (0.997–1.016)	—
4 years	2.7% (1.4%–4.0%)	1.011 (0.999–1.023)	466
5 years	4.0% (2.4%–5.7%)	1.002 (0.986–1.018)	—
6 years	4.5% (2.7%–6.3%)	1.006 (0.985–1.027)	328
7 years	6.1% (3.9%–8.4%)	1.006 (0.983–1.028)	—
8 years	6.5% (4.1%–8.8%)	1.010 (0.982–1.038)	220
9 years	6.9% (4.4%–9.4%)	1.018 (0.988–1.049)	—
10 years	7.5% (4.8–10.3%)	1.023 (0.982–1.065)	129

Relative survival estimated using the Pohar–Perme method against Czech population life tables matched on age, sex and calendar year. A ratio of 1.0 indicates equivalence with population expectation; values whose 95% CIs span 1.0 are consistent with no excess mortality.

Numbers at risk are reported at baseline and at two-year intervals. Em dashes indicate time points for which numbers at risk were not separately reported.

### 3.9 Cause-specific mortality

**Figure 7:**
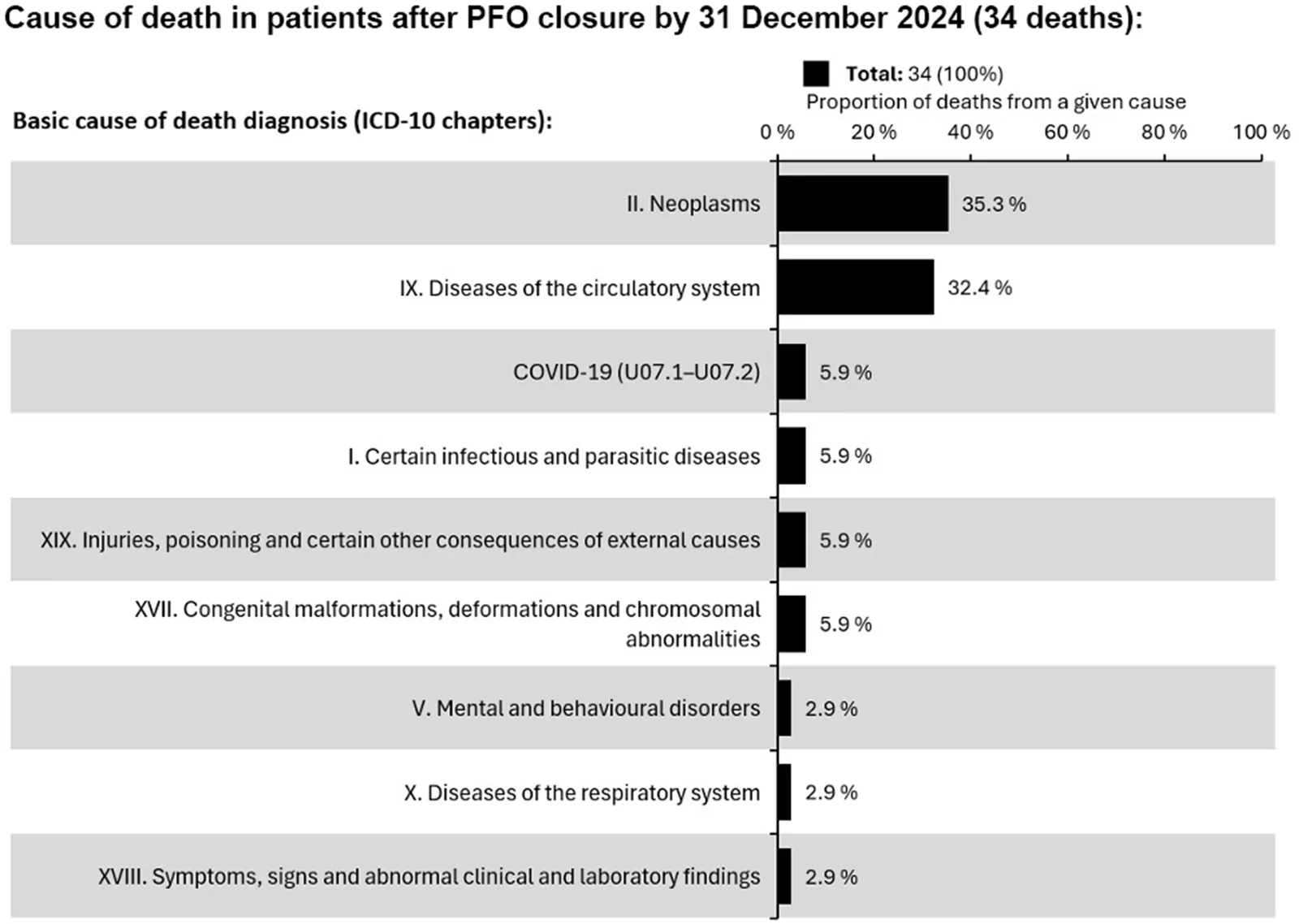
Distribution of causes of death according to ICD-10 chapter classification. Cause-specific mortality among patients undergoing percutaneous PFO closure between 2010 and 2024 (*n* = 34 deaths). Causes of death were classified according to ICD-10 chapter categories derived from the Czech Statistical Office mortality database.

Among the 34 deaths recorded during follow-up, the most frequent primary cause was neoplasm (ICD-10 chapter II), accounting for 12 deaths (35.3%). Diseases of the circulatory system (chapter IX) accounted for 11 deaths (32.4%). COVID-19 (U07.1–U07.2) was the primary cause in 2 deaths (5.9%). Infectious and parasitic diseases (chapter I), external causes (chapter XIX) and congenital malformations (chapter XVII) each accounted for 2 deaths (5.9%), while mental and behavioural disorders (chapter V), respiratory diseases (chapter X) and ill-defined causes (chapter XVIII) each accounted for one death (Table 8, Figure 7). No death was coded as procedure-related.

**Table 8:** Distribution of primary causes of death by ICD-10 chapter in the PFO closure cohort (*n* = 34 deaths).

Primary cause of death (ICD-10 chapter)	*n* (%)
II. Neoplasms	12 (35.3%)
IX. Diseases of the circulatory system	11 (32.4%)
U07.1–U07.2. COVID-19	2 (5.9%)
I. Certain infectious and parasitic diseases	2 (5.9%)
XIX. Injuries, poisoning and other external causes	2 (5.9%)
XVII. Congenital malformations and chromosomal abnormalities	2 (5.9%)
V. Mental and behavioural disorders	1 (2.9%)
X. Diseases of the respiratory system	1 (2.9%)
XVIII. Symptoms, signs and abnormal clinical and laboratory findings	1 (2.9%)
**Total**	**34** (**100%)**

Classified by primary cause of death using ICD-10 chapter as recorded in the Czech Statistical Office (ČSÚ) database of deceased persons.

Bold values indicate totals.

## 4 Discussion

### 4.1 Principal findings

In this retrospective single-centre cohort study of 707 patients undergoing percutaneous PFO closure between 2010 and 2024 at the Cardiology Department of Motol and Homolka University Hospital, Homolka Campus, Prague, Czech Republic, with deterministic linkage to nationwide hospitalisation, pharmacy-dispensing and mortality registries, we observed three principal findings.

First, the crude proportion of patients with a clinically recognised, hospital-recorded AA over a maximum follow-up of 14.9 years was low (1.84%; 10-year Kaplan–Meier cumulative incidence 2.9%, 95% CI 1.2%–4.6%), with some events occurring early and, notably, almost half occurring more than three years after closure.

Second, patients who developed AA were predominantly male and had a high baseline cardiovascular risk burden, with higher observed proportions of HTN, HLP and DM than in the parent cohort (Table 5).

Third, 10-year observed survival closely matched age-, sex- and calendar year-adjusted Czech population life-table expectations; relative survival was close to unity throughout follow-up and was not significantly below unity at any time point, indicating no observed excess mortality.

These three observations are descriptive; given the small number of arrhythmic events (*n* = 13), the temporal and comorbidity patterns should be regarded as hypothesis-generating rather than definitive.

### 4.2 Long-term arrhythmic burden in context

Our observed long-term cumulative incidence of AA (2.9% at 10 years, 95% CI 1.2%–4.6%; 13 events over 4,329 patient-years, corresponding to an incidence rate of 3.0 per 1,000 patient-years) lies at the lower end of the range reported in the literature when ascertainment is restricted to clinically attended, ICD-10-coded hospital events. A nationwide Danish cohort of 817 patients undergoing PFO closure between 2008 and 2020 reported an approximately 12% 10-year cumulative incidence of AF or AFL (21); however, that cohort included patients with prior arrhythmic episodes and used both inpatient and outpatient specialist contact codes, broadening the case definition. A 15-year Ontario provincial linkage of 2,750 patients reported an unadjusted incidence rate of new-onset AF of 18.6 per 1,000 person-years (22), substantially higher than our estimate. Differences between these estimates may reflect variation in patient selection, administrative coding algorithms, endpoint definitions and surveillance intensity and should not be interpreted as evidence of a true difference in arrhythmic burden. Reported incidence varies widely across observational cohorts according to surveillance strategy, endpoint definition and follow-up duration (11, 13, 15, 18). Our findings are therefore broadly consistent with the observational evidence base, with the important caveat that hospital-based ascertainment captured clinically recognised, hospital-recorded AA events rather than the total arrhythmic burden. Asymptomatic, brief paroxysmal episodes and events managed exclusively in primary care may not have been recorded.

### 4.3 Late events and the natural-history question

A notable descriptive observation of our study may not be the headline incidence figure, but the temporal distribution of events. Of the 13 events, only two occurred within the conventional 90-day peri-procedural window, contrasting with the well-described early peak reported in randomised trials and prolonged-monitoring studies (8–13). Six events (46.2%) occurred more than three years after closure. This late occurrence raises an important mechanistic question.

Two non-exclusive explanations merit consideration. The first is residual device- or substrate-related atrial remodelling. Several mechanisms have been proposed to explain arrhythmia after closure, predominantly in the early period (8, 28). Mechanical factors include direct irritation and stretch of the interatrial septum by the implanted device and possible disruption of interatrial conduction through the Bachmann bundle near the superior margin of the fossa ovalis, with device-related changes in P-wave duration described after septal device closure (29). The device also elicits a foreign-body inflammatory response—comprising pro-inflammatory, proliferative and endothelialisation/resolution phases—in which cytokines such as TNF-α and IL-6 promote fibrosis, gap-junction and calcium-handling abnormalities and sympathetic activation, generating a transient arrhythmogenic substrate (30, 31). Transient nickel release from the nitinol frame before complete endothelialisation (typically within one to three months) and type IV (nickel) hypersensitivity have additionally been proposed, temporally coinciding with the early arrhythmic peak, although a definite arrhythmic effect remains unproven (28). Finally, closure eliminates a small left-to-right shunt and alters left atrial mechanics, with speckle-tracking studies demonstrating early reductions in left atrial contractile strain and increases in left atrial volume that may transiently lower the threshold for AA (32–34). These mechanisms predominantly account for early, peri-procedural events; their relevance to the predominantly late-onset arrhythmias observed in our cohort is uncertain. The second explanation is that these late-onset events largely reflect the background incidence of age- and comorbidity-related atrial tachyarrhythmias (AF, AFL and AT) that would be expected in a comparable population irrespective of PFO closure, with the closure device acting principally as a temporal landmark rather than a causal driver. In other words, the late arrhythmias observed during follow-up may reflect the natural history of AA in an ageing, risk-factor-enriched cohort rather than a device-specific effect.

### 4.4 Comorbidity-driven arrhythmic risk

The marked enrichment of HTN, HLP and DM among the 13 patients with AA, when contrasted with the parent cohort (Table 5), is consistent with the second explanation above. HTN, HLP and DM are, together with older age, well-established risk factors for atrial tachyarrhythmias, as summarised in large epidemiological cohorts and the 2024 ESC guidelines on AF (35). Male predominance among the affected patients is consistent with the higher age-adjusted incidence of atrial fibrillation reported in men. The high baseline prevalence of HTN and HLP in our cohort is consistent with the cardiovascular epidemiology of the Czech population, which is classified as a high-risk region within the ESC SCORE2 framework (36) and has a high background prevalence of these conditions. Because HLP was operationally defined by dispensing of lipid-modifying therapy, its high recorded prevalence may partly reflect secondary-prevention treatment following stroke or TIA rather than independently documented HLP. Observations involving HLP should therefore be interpreted cautiously. In the absence of a comparator group, however, the observed late arrhythmias cannot be causally attributed to PFO closure and cannot be distinguished from the arrhythmia incidence that would be expected in a similar population without an occluder. This descriptive pattern is compatible with background cardiovascular risk but does not identify a validated high-risk subgroup or establish that differential rhythm surveillance improves outcomes. Prospective evaluation is required.

### 4.5 Related pharmacotherapy

The pharmacy-dispensing data captured by NRHZS provide a population-level view of antithrombotic practice after PFO closure. Antiplatelet therapy with acetylsalicylic acid and clopidogrel remained the dominant post-procedural strategy throughout the study period. Anticoagulant prescribing evolved over time, with vitamin K antagonist use declining and DOACs becoming more frequent in later years. Although the present study was not designed to compare the efficacy or safety of specific antithrombotic regimens, these dispensing patterns provide relevant real-world context for interpretation of the observed arrhythmic and mortality outcomes.

### 4.6 Catheter ablation feasibility

Catheter ablation for AA was performed in a very limited number of patients, with a 10-year cumulative incidence of 2.0%. This finding is consistent with published data and supports the accumulated clinical experience that transseptal puncture and pulmonary vein isolation remain feasible in patients with previously implanted PFO closure devices, either through the device or adjacent to it depending on anatomy and operator strategy (26, 27). Several different occluder types were implanted over the study period, and no device-related procedural failure was documented at our centre.

### 4.7 Survival in a real-world setting

Perhaps the most reassuring finding of the present analysis is the absence of any excess mortality compared with the matched Czech general population. To our knowledge, this is the first PFO closure cohort to apply the Pohar–Perme relative survival estimator. Importantly, relative survival was not significantly below unity at any time point; where it differed significantly from unity (at 1 and 2 years) it lay marginally above 1.0—a pattern compatible with the selection of relatively healthy individuals (those judged fit for percutaneous intervention) into the cohort. The leading attributed causes of death were neoplasms (35.3%) and cardiovascular disease (32.4%); no death was coded as procedure-related. The two COVID-19 deaths reflect the impact of the pandemic on the cohort during the 2020–2022 calendar window.

### 4.8 Strengths and limitations

The principal strength of this analysis is the deterministic linkage of a single-centre institutional PFO closure registry to nationwide hospitalisation, pharmacy-dispensing and mortality databases through the universal Czech personal identifier. This enabled near-complete long-term follow-up and rendered loss to follow-up effectively negligible. A further methodological strength is the application of the Pohar–Perme relative survival estimator against age-, sex- and calendar year-matched Czech population life tables, providing a population-standardised survival benchmark not previously applied in a PFO closure cohort.

Several limitations warrant consideration. First, outcome ascertainment was restricted to hospital-recorded arrhythmias. Asymptomatic, self-limited episodes and AA managed exclusively in primary care or outside hospital-based specialist services may therefore have been missed. The reported incidence reflects clinically recognised, hospital-recorded events rather than the total arrhythmic burden. Continuous-monitoring studies have reported substantially higher detection rates, with ICM data suggesting 10%–37% AF detection within the first year (12, 15, 16), although the clinical significance of many brief, asymptomatic episodes remains uncertain.

Second, only 13 qualifying arrhythmic events were observed. Comparisons between affected patients and the parent cohort, as well as the temporal distribution of events, are therefore descriptive and hypothesis-generating. Detailed procedural variables, including device size, residual shunt and peri-procedural rhythm-monitoring duration, were unavailable in the linked analytical dataset. Exact patient-level counts according to indication subtype were also unavailable. Device-level data were likewise not included, and a device-stratified comparison would not have been statistically meaningful with only 13 events. Although differing arrhythmia rates across occluders have been reported (28), available comparisons are predominantly non-randomised and involve heterogeneous populations; our study cannot address device-specific arrhythmic risk.

Third, the study was single-centre with respect to the index procedure, which may limit generalisability to lower-volume centres. Follow-up duration also varied substantially across the accrual period: patients treated in the earliest years contributed up to 14.9 years, whereas those treated in 2023–2024 contributed only months. Consequently, later procedural cohorts contributed little person-time to the long-term estimates, the number of patients at risk decreased at later time points, and the 10-year estimates are driven predominantly by earlier cohorts. These estimates should therefore be interpreted cautiously and may not fully reflect outcomes with the most contemporary device generations.

Fourth, the study lacked a matched comparator group of patients without PFO closure. The arrhythmias observed during follow-up therefore cannot be causally attributed to the procedure. In particular, late events among patients with a substantial baseline cardiovascular risk burden cannot be distinguished from the background incidence expected in a comparable population without an occluder.

Fifth, recurrent ischaemic stroke or TIA could not be reliably ascertained because post-closure administrative diagnoses may have represented carry-forward coding of the index cerebrovascular event and were neither adjudicated against source records nor identified using a validated acute-event algorithm. Recurrent cerebrovascular events were not a prespecified endpoint and are the subject of planned future analyses.

Finally, although the absence of documented AA and a negative pre-closure Holter assessment were prerequisites for closure, a pre-closure administrative arrhythmia code was present in 12 patients. These codes could not be adjudicated against source electrocardiographic recordings and may have represented provisional or non-specific coding, such as brief atrial runs or supraventricular ectopy, rather than established AA. Only one of these patients subsequently had a post-closure event. These pre-closure codes were identified from unfiltered historical administrative records, whereas the post-closure endpoint required a qualifying hospital-based encounter with AA recorded as a primary or secondary diagnosis. This higher ascertainment threshold reduces susceptibility to incidental, provisional or non-specific coding and increases the clinical specificity of the post-closure endpoint. Conversely, episodes that did not prompt hospital-based evaluation—particularly asymptomatic, brief or self-limited events—would not have been captured. The dominant ascertainment limitation is therefore under-detection of total arrhythmic burden rather than inflation by isolated diagnostic codes. As administrative codes could not be adjudicated against source electrocardiographic recordings, the endpoint is reported as the first hospital-recorded AA after closure rather than as strictly incident or *de novo* disease.

### 4.9 Clinical implications and future directions

Several clinically relevant implications emerge from our findings.

First, the long-term burden of clinically recognised AA events after percutaneous PFO closure appears low in routine real-world practice, but not negligible, and extends substantially beyond the early peri-procedural period. Surveillance strategies focused exclusively on the first 30–45 days after closure are therefore likely to fail to capture clinically attended late arrhythmic events.

Second, the higher observed prevalence of HTN, HLP and DM among patients with post-closure AA provides a rationale for prospective evaluation of risk-stratified follow-up. These descriptive observations support prospective evaluation of risk-factor-based rhythm-surveillance strategies but do not establish that intensified long-term monitoring improves clinical outcomes. Whether such patients benefit from more intensive post-closure rhythm surveillance is the subject of our ongoing prospective study (ClinicalTrials.gov NCT06983613). For pre-closure rhythm assessment and post-closure medical management, current European guidance should be followed (6, 25).

Third, the absence of observed excess mortality relative to the age-, sex- and calendar year-matched Czech general population life-table expectations provides reassurance regarding the long-term safety of percutaneous PFO closure when performed in experienced centres.

Future research should focus on prospective multicentre registries integrating continuous rhythm monitoring with nationwide administrative follow-up in order to reconcile the discrepancy between subclinical and clinically manifest arrhythmic burden after closure. Standardisation of outcome definitions across electrophysiological studies, clinical registries and administrative datasets will be essential for meaningful comparison between cohorts. Further investigation into imaging-, biomarker- and substrate-based predictors of late atrial arrhythmogenesis may enable development of personalised post-closure surveillance strategies in this expanding patient population.

## 5 Conclusion

In this retrospective single-centre cohort linked deterministically to nationwide Czech hospitalisation, pharmacy-dispensing and mortality registries, the long-term incidence of clinically recognised AA events following percutaneous PFO closure was low but not negligible (10-year cumulative incidence 2.9%), with most events occurring beyond the immediate peri-procedural period and predominantly in patients with substantial baseline cardiovascular risk burden. Given the small number of arrhythmic events, these temporal and comorbidity patterns are hypothesis-generating and require confirmation in larger prospective cohorts. Catheter ablation for post-closure atrial tachyarrhythmia was uncommon and was performed without documented device-related procedural limitation or failure. Long-term observed survival closely matched that expected for the age-, sex- and calendar year-matched Czech general population, with no observed excess mortality relative to population life-table expectations. The mortality findings provide reassurance regarding long-term outcomes, whereas the arrhythmic observations support prospective evaluation of risk-stratified surveillance rather than an immediate change in clinical practice.

## Data Availability

The datasets analysed for this study are not publicly available because they contain pseudonymised individual-level information derived from Czech national administrative health registries, which cannot be shared under national legislation (Act No. 372/2011 Coll.). Aggregated data are available from the corresponding author on reasonable request and, where applicable, on the basis of a data-access agreement with the Institute of Health Information and Statistics of the Czech Republic (ÚZIS ČR).
