# Supplementary material for: Long-term incidence of first hospital-recorded atrial arrhythmias and all-cause mortality after percutaneous patent foramen ovale closure: a single-centre cohort linked to Czech nationwide administrative health data
Source: Front Cardiovasc Med. 2026 Aug 25;13:1896548. doi: 10.3389/fcvm.2026.1896548 (PMC13550008; doi:10.3389/fcvm.2026.1896548)
Supplement: Supplementary file 1 [file Supplementaryfile1.docx]

Long-Term Incidence of First Hospital-Recorded Atrial Arrhythmias and All-Cause Mortality After Percutaneous Patent Foramen Ovale Closure: A Single-Centre Cohort Linked to Czech Nationwide Administrative Health Data – supplementary material

Table S1. *Annual prevalence of cardiovascular comorbidities among patients undergoing PFO closure, 2010–2024.*

| **Year** | **Patients** | **DM, n (%)** | **HTN, n (%)** | **HLP, n (%)** |
| --- | --- | --- | --- | --- |
| 2010 | 17 | 1 (5.9%) | 12 (70.6%) | 7 (41.2%) |
| 2011 | 26 | 3 (11.5%) | 15 (57.7%) | 9 (34.6%) |
| 2012 | 15 | 4 (26.7%) | 10 (66.7%) | 7 (46.7%) |
| 2013 | 35 | 3 (8.6%) | 16 (45.7%) | 22 (62.9%) |
| 2014 | 48 | 6 (12.5%) | 31 (64.6%) | 24 (50.0%) |
| 2015 | 49 | 3 (6.1%) | 32 (65.3%) | 37 (75.5%) |
| 2016 | 46 | 6 (13.0%) | 34 (73.9%) | 39 (84.8%) |
| 2017 | 49 | 12 (24.5%) | 29 (59.2%) | 36 (73.5%) |
| 2018 | 59 | 12 (20.3%) | 37 (62.7%) | 45 (76.3%) |
| 2019 | 73 | 10 (13.7%) | 45 (61.6%) | 50 (68.5%) |
| 2020 | 62 | 9 (14.5%) | 39 (62.9%) | 44 (71.0%) |
| 2021 | 62 | 9 (14.5%) | 36 (58.1%) | 49 (79.0%) |
| 2022 | 55 | 9 (16.4%) | 39 (70.9%) | 40 (72.7%) |
| 2023 | 63 | 9 (14.3%) | 33 (52.4%) | 48 (76.2%) |
| 2024 | 48 | 10 (20.8%) | 32 (66.7%) | 36 (75.0%) |
| **Total** | **707** | **106 (15.0%)** | **440 (62.2%)** | **493 (69.7%)** |

*DM, diabetes mellitus; HLP, hyperlipidaemia; HTN, arterial hypertension. See Materials and methods, section 2.6 for operational definitions.*

*Percentages are calculated using the number of unique patients undergoing PFO closure within each calendar year as the denominator.*

Table S2. *Annual dispensing of antithrombotic medications within 90 days after PFO closure, 2010–2024.*

| **Year** | **Patients** | **Acetylsalicylic acid (B01AC06)** | **Clopidogrel (B01AC04)** | **Warfarin (B01AA03)** | **DOACs† combined** |
| --- | --- | --- | --- | --- | --- |
| 2010 | 17 | 12 (70.6%) | 15 (88.2%) | 3 (17.6%) | 0 (0.0%) |
| 2011 | 26 | 24 (92.3%) | 24 (92.3%) | 2 (7.7%) | 0 (0.0%) |
| 2012 | 15 | 9 (60.0%) | 11 (73.3%) | 2 (13.3%) | 0 (0.0%) |
| 2013 | 35 | 30 (85.7%) | 33 (94.3%) | 3 (8.6%) | 0 (0.0%) |
| 2014 | 48 | 43 (89.6%) | 47 (97.9%) | 4 (8.3%) | 0 (0.0%) |
| 2015 | 49 | 37 (75.5%) | 47 (95.9%) | 2 (4.1%) | 2 (4.1%) |
| 2016 | 46 | 38 (82.6%) | 38 (82.6%) | 2 (4.3%) | 3 (6.5%) |
| 2017 | 49 | 39 (79.6%) | 44 (89.8%) | 1 (2.0%) | 4 (8.2%) |
| 2018 | 59 | 46 (78.0%) | 55 (93.2%) | 0 (0.0%) | 3 (5.1%) |
| 2019 | 73 | 58 (79.5%) | 68 (93.2%) | 0 (0.0%) | 4 (5.5%) |
| 2020 | 62 | 47 (75.8%) | 56 (90.3%) | 0 (0.0%) | 8 (12.9%) |
| 2021 | 62 | 49 (79.0%) | 55 (88.7%) | 1 (1.6%) | 9 (14.5%) |
| 2022 | 55 | 46 (83.6%) | 50 (90.9%) | 1 (1.8%) | 4 (7.3%) |
| 2023 | 63 | 51 (81.0%) | 57 (90.5%) | 0 (0.0%) | 6 (9.5%) |
| 2024 | 48 | 36 (75.0%) | 41 (85.4%) | 0 (0.0%) | 4 (8.3%) |

*Values are presented as counts and percentages of unique patients undergoing PFO closure in the respective calendar year who were dispensed the given medication within the 90-day post-procedural window.*

*†DOACs combined column represents the total number of patients dispensed apixaban (B01AF02), rivaroxaban (B01AF01), dabigatran (B01AE07) or edoxaban (B01AF03). Individual patients may appear in more than one medication category.*

*ATC codes for antiplatelet therapy: B01AC04, clopidogrel; B01AC05, ticlopidine; B01AC06, acetylsalicylic acid; B01AC10, indobufen; B01AC13, abciximab; B01AC21, treprostinil; B01AC22, prasugrel; B01AC23, cilostazol; B01AC24, ticagrelor; B01AC25, cangrelor; B01AC30, combinations.*

*ATC codes for anticoagulant therapy: B01AA03, warfarin; B01AB01, heparin; B01AB02, antithrombin III; B01AB04, dalteparin; B01AB05, enoxaparin; B01AB06, nadroparin; B01AB11, sulodexide; B01AB12, bemiparin; B01AE07, fondaparinux; B01AX05, dabigatran; B01AF01, rivaroxaban; B01AF02, apixaban; B01AF03, edoxaban.*
